# Rapid Alternate Monocular Deprivation Does Not Affect Binocular Balance and Correlation in Human Adults

**DOI:** 10.1523/ENEURO.0509-21.2022

**Published:** 2022-05-17

**Authors:** Wenman Lin (林温曼), Junhan Wei (魏君涵), Wenjing Wang (王文静), Liying Zou (邹李颖), Shiqi Zhou (周诗旗), Nan Jiang (江楠), Alexandre Reynaud, Jiawei Zhou (周佳玮), Xudong Yu (于旭东), Robert F. Hess

**Affiliations:** 1 School of Ophthalmology and Optometry and Eye hospital, and State Key Laboratory of Ophthalmology, Optometry and Vision Science, Wenzhou Medical University, Wenzhou 325000, People’s Republic of China; 2 Xi'an People's Hospital (Xi'an Fourth Hospital), Shaanxi Eye Hospital, Affiliated Guangren Hospital School of Medicine, Xi'an Jiaotong University, Xi'an 710004, People’s Republic of China; 3 McGill Vision Research, Department of Ophthalmology & Visual Sciences, McGill University, Montreal, Québec H4A 3S5, Canada

**Keywords:** binocular balance, flicker, interocular correlation, visual deprivation, visual plasticity

## Abstract

Recent studies show that the human adult visual system exhibits neural plasticity. For instance, short-term monocular deprivation shifts the eye dominance in favor of the deprived eye. This phenomenon is believed to occur in the primary visual cortex by reinstating neural plasticity. However, it is unknown whether the changes in eye dominance after monocularly depriving the visual input can also be induced by alternately depriving both eyes. In this study, we found no changes in binocular balance and interocular correlation sensitivity after a rapid (7 Hz), alternate, and monocular deprivation for 1 h in adults. Therefore, the effect of short-term monocular deprivation cannot seem to be emulated by alternately and rapidly depriving both eyes.

## Significance Statement

Previous work has shown that short-term binocular function disruption, which in its most extreme form is monocular deprivation, could induce neural plasticity in the adult visual system. In this study, we found that a balanced deprivation of binocular function could not induce a neuroplastic change in human adults. It appears that ocular dominance plasticity in human adults is unique in so far as it is only driven by an input imbalance not balanced deprivation of binocular function.

## Introduction

Monocular deprivation during the early critical period of visual development dramatically alters the neural circuitry in regions of the primary visual cortex that process binocular visual information. For example, monocular deprivation (i.e., lid suture) in kittens (Hubel and Wiesel, 1964) and monkeys (Hubel et al., 1977) can permanently blind the deprived eye. While this manipulation has not been shown to produce any permanent disruption in the adult visual system because it exhibits less neural plasticity (Hubel and Wiesel, 1970; Berardi et al., 2000).

However, recent studies have revealed that the adult visual system displays a more limited and temporary form of neural plasticity. For instance, 2.5 h of short-term monocular deprivation in the adult results in an increase in the contribution of the deprived eye in binocular vision (Lunghi et al., 2011). Such an effect can last for about 30 min after the deprivation is discontinued. This finding has been corroborated using psychophysical methods, such as binocular combination (Zhou et al., 2013a) and binocular rivalry (Lunghi et al., 2011). Short-term monocular deprivation can also affect both achromatic and chromatic binocular processes (Lunghi et al., 2013; Lunghi and Alais, 2013; Zhou et al., 2017a, b). Not only can changes in sensory eye balance in the adult be induced by depriving the visual content of one eye, but they can also occur by selectively depriving the visual information of one eye. To illustrate, the removal of high spatial frequency information (Zhou et al., 2014b) and orientational information (Zhou et al., 2014b; Wang et al., 2017) can disrupt binocular balance. Apart from the above psychophysical studies, neural imaging studies, with event-related potentials (Lunghi et al., 2015b), steady-state visual evoked potentials (Zhou et al., 2015; Chadnova et al., 2017), functional magnetic resonance spectroscopy (Lunghi et al., 2015a), and 7 T functional magnetic resonance imaging (Binda et al., 2017) show that the primary visual cortex involves in these neuroplastic changes.

Recently, it has been suggested (Zhou et al., 2013c) that this neuroplastic change could provide a novel binocular treatment for amblyopia, which has shown promise in two laboratory clinical studies where binocular and monocular benefits result from the patching of the amblyopic eye in adults (for 2–3 h/d for ≥2 months; Zhou et al., 2019; Castaldi et al., 2020).

In summary, binocular function can be perturbed by partially or entirely depriving one eye. However, whether a binocular imbalance is necessary for shifting eye dominance by inducing neural plasticity is unknown. In fact, an entirely balanced disruption to binocular input exists because of the advent of technology. One can be subjected to an alternate deprivation of each eye at 7 Hz so that binocular visual input is disrupted in a balanced fashion. In this study, we examine whether alternate deprivation for 1 h can also emulate the effect of short-term monocular deprivation by quantifying the binocular balance and interocular correlation sensitivity (i.e., sensitivity of binocular function) of normally sighted individuals. Specifically, the binocular balance was measured using a binocular phase combination paradigm that measures the relative contribution of each eye to binocular function. This measurement was first introduced by Ding and Sperling (2006) and has been widely used in measuring binocular balance. Binocular perception relies on the internal representations of the inputs of two eyes. When binocular balance favors one eye, the visual input from said eye can dominate over binocular perception, thereby resulting in a binocular vision that heavily weighs the input of said eye. This imbalance can be quantified by using the binocular combination paradigm. In addition, we used a quick correlation sensitivity function approach, which was introduced by Reynaud and Hess (2018) to assess the interocular correlation sensitivity of binocular vision. Interocular correlation is defined as the degree to which the images in the two eyes match one another (i.e., the degree to which the information in the two eyes correlates). In our study, we alternately deprived both eyes so that balanced, but monocular, visual stimuli could be presented. When each eye gets deprived alternatively, interocular correlation could be disrupted. We wonder whether this persistent disruption in interocular correlation at a rapid frequency would affect binocular function through neuroplasticity. To ascertain these questions. we needed these two measurements, one involving the lower-level combination and the other the degree of interocular correlation.

## Materials and Methods

### Participants

Ten adults (average age: 27.00 ± 1.00 years; six females) with normal or corrected-to-normal monocular visual acuity [no worse than 0.0 logMAR (logarithm of the minimum angle of resolution)] participated in this study. Participants were required to wear their habitual optical correction [refractive errors ranging from Plano to −7.75 diopters (D)]. All case patients had no history of deprivation therapy, visual training, or ocular surgeries. The sample size has been chosen based on previous studies of monocular deprivation (Zhou et al., 2017a, b; Min et al., 2018) using a similar binocular phase combination measure as the current study. For example, Min et al. (2018) found that 1 h of monocular deprivation induces an immediate patching effect of 1.39 ± 1.24 dB (i.e., change in contrast balance ratio at the 60 min patching condition; their Fig. 2b; mean ± SD). With a power of 80% and α-level of 0.05, we found that the minimum sample size would have to be seven subjects. Moreover, Zhou et al. (2017a) found that 2 h of monocular deprivation induces an immediate patching effect of 6.3 ± 3.3° (i.e., perceived phase change at the rest condition; their Fig. 2c); Zhou et al. (2017b) found that 2.5 h of monocular deprivation induces an immediate patching effect of 19.2 ± 7.0° (i.e., perceived phase change tested with achromatic gratings; their Fig. 1c and supplementary). We found that a sample size of three subjects would be enough to get a power of 80% and α-level of 0.05 based on these two reports. To make our study robust, we recruited 10 subjects into this study.

All participants were naive to the purpose of the study and provided informed consent before the study. This study was performed in accordance with the tenets of the Declaration of Helsinki and was approved by the Ethics Committee of the Wenzhou medical university.

### Apparatus

We performed alternative monocular deprivation at 7 Hz using the Eyetronix Flicker Glasses (Eyetronix; http://eyetronix.com). The product consisted of portable eyeglasses with liquid crystal lenses, which could be controlled electronically to flicker at a specific temporal frequency (e.g., 7 Hz). In this study, the glass was preprogrammed to produce rapid, organized, and direct square-wave deprivation with a 50/50 flicker alternation rate at 7 Hz.

Binocular balance measurements were performed on a Macintosh computer equipped with MATLAB (MathWorks) and PsychToolBox 3.0.9 extensions (Brainard, 1997; Pelli, 1997). We dichoptically displayed the stimuli using γ-corrected head-mounted 3D goggles (Goovis Pro, NED Optics). The refresh rate of the display was 60 Hz in each eye, and the resolution was 1600 × 900 pixels (corresponding to 46° × 26°). The maximal luminance of the OLED (organic LED) goggles was 150 cd/m^2^.

Interocular correlation measurements were conducted using a PC computer (Alienware Area-51 R2). We programmed the test that measures binocular balance using MATLAB R2016b (MathWorks) and the Psychophysics Toolbox (Brainard, 1997; Pelli, 1997). All stimuli were presented on a γ-corrected 3D LCD screen (ROG Swift PG278QR, ASUS), which had a resolution of 2560 × 1440 pixels with a refresh rate of 165 Hz and used the G-Sync technology (ASUS). The subject viewed the stimuli in a dim-lit room, at a viewing distance of 70 cm, with active, shutter 3D glasses (3D VISION2 P1431, NVIDIA).

### Design

**Figure 1.**
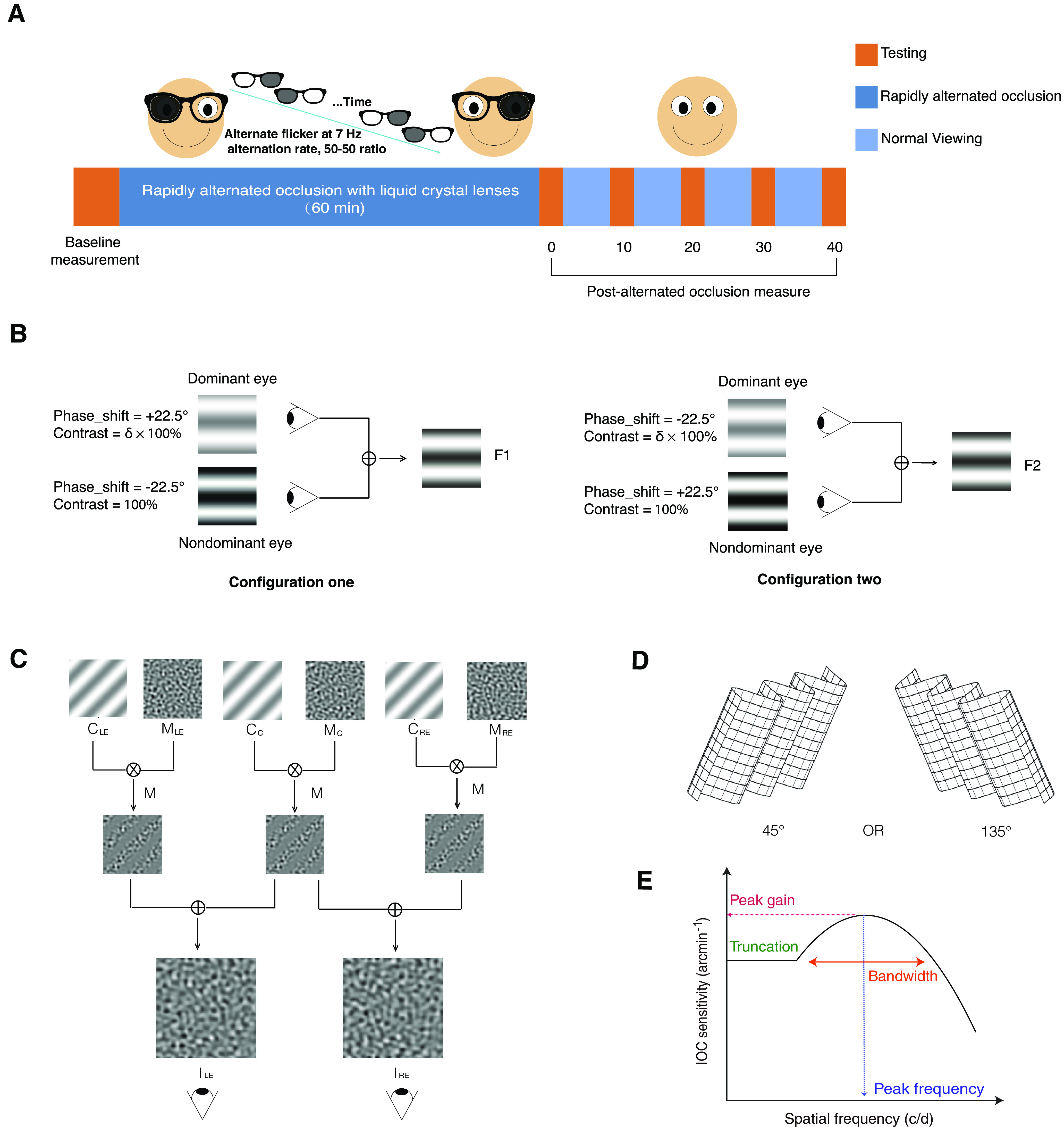
Illustration of the experimental design. ***A***, Observers wore Eyetronix Flicker Glass (EFG) for 1 h. Their binocular balance and interocular correlation function were assessed during one pre-deprivation session and five post-deprivation sessions. Post-deprivation sessions were run at 0, 10, 20, 30, and 40 min after deprivation. ***B***, Binocular balance measurement. The images viewed by subjects were composed of two horizontal sine-wave gratings with equal and opposite phase shifts of 22.5°. The stimulus contrast for the nondominant eye was fixed at 100% and the dominant eye was δ × 100%. δ is the interocular contrast ratio that was selected in practice trials. To avoid potential positional errors, in configuration one, the phase of the dominant eye was set as +22.5°, and the other eye was set as −22.5° and used in reverse in configuration two. ***C***, Interocular correlation measurement. The images for the two eyes were composed of two blended filtered noise textures C_C_ (the common one that constituted the correlated part of the stimulus) and C_LE_ or C_RE_, respectively (the uncorrelated part for the left eye and the right eye), modulated by out-of-phase sinusoidal envelopes *M*_C_, *M*_LE_, and *M*_RE_ of one-quarter the frequency of the noise pattern (*M*_LE_ is the same as *M*_RE_). And we could adjust the modulation parameter *M* to change the amount of correlation present in the common stripes of two eyes. Thus, those who have a degree of interocular correlation could see a visual effect of luster with orientation at 45° or 135° (here at 135°). ***D***, The subject task was to identify the orientation of the correlation modulation of the stimulus, which could be oblique at 45° (left) or 135° (right). ***E***, The interocular correlation (IOC) sensitivity function is described as a function of the spatial frequency by the truncated log-parabola model. Four parameters are studied: the peak gain, the peak frequency, the bandwidth, and the truncation.

Two experiments using two different binocular measures, binocular balance and interocular correlation, were conducted on separate days with a randomized order across subjects. Each experiment consisted of the following three consecutive stages (Fig. 1*A*): a pre-deprivation measurement (binocular balance or interocular correlation); 1 h of 7 Hz alternate monocular deprivation stage; and a post-deprivation measurement. During the deprivation stage, the Eyetronix Flicker Glass was used to produce a 50/50 flicker alternation rate between the two eyes at 7 Hz. In each experiment, we tested the changes of binocular balance or interocular correlation before and after deprivation sessions (0, 10, 20, 30, and 40 min) to quantify the effect of 1 h of alternate monocular deprivation. Before the start of measurement, the participants were asked to finish practice trials to ensure that they had understood the tasks. During the period of deprivation, which involved alternate flicker stimulation, participants were allowed to perform their daily activities such as reading books, walking around or using electronic devices such as cell phones and laptops.

#### Binocular balance measurement

Binocular balance was quantitatively assessed using a binocular phase combination paradigm (Ding and Sperling, 2006). In this measure, two horizontal sine-wave gratings with an equal and opposite phase shift of 22.5° were dichoptically viewed by participants. We measured the perceived phase of the grating in the cyclopean percept at an interocular contrast ratio (δ) where participants’ two eyes had a balanced contribution (zero phase cyclopean percept) before deprivation. During one trial, the perceived phases were measured when the phase shift of the grating of the dominant eye was +22.5° and that of the nondominant eye was −22.5° (Fig. 1*B*, configuration one) and vice versa (Fig. 1*B*, configuration two) to avoid potential positional errors. Each configuration was tested eight times with the method of constant stimuli. In all, there were 16 trials in one measurement that lasted about 3 min each (2 configurations × 8 repetitions). If, consequently, the contribution of the dominant eye to binocular vision increased after deprivation, the binocular perceived phase became more negative; otherwise, if it decreased, the binocularly perceived phase became more positive.

#### Interocular correlation measurement

The interocular correlation sensitivity was assessed using a quick correlation sensitivity function approach (Reynaud and Hess, 2018), adapted from a quick contrast sensitivity function (qCSF) method (Hou et al., 2010; Lesmes et al., 2010). The interocular sensitivity as a function of stimulus spatial frequency was measured with a log-parabola model, which is described by four parameters [Fig. 1*E*; the peak frequency (*f*_max_), the peak gain (γ_max_), the bandwidth (β), and the truncation (δ; Eq. 1; Ahumada and Peterson, 1992; Watson and Ahumada, 2005; Lesmes et al., 2010], as follows:

$$S^{\prime }\left(f\right)={\text{log}}_{10}\left(\gamma_{\text{max}}\right)-\kappa {\left(\frac{{\text{log}}_{10}\left(f\right)-{\text{log}}_{10}(f_{\text{max}})}{\beta^{\prime }/2}\right)}^{2},$$

if

$$f<f_{\text{max}}\text{ and }S^{\prime }\left(f\right)<{\text{log}}_{10}\left(\gamma_{\text{max}}\right)-\delta ,$$

then

$$S\left(f\right)={\text{log}}_{10}\left(\gamma_{\text{max}}\right)-\delta ,$$

otherwise

(1) $$S(f)=S^{\prime }\left(f\right).$$

The two images were viewed dichoptically using a polarized 3D screen. They were composed of two blended filtered noise textures C_C_ (the common one, which constitutes the correlated part of the stimulus) and C_LE_ or C_RE_, respectively (the uncorrelated part for the left eye and the right eye), which were modulated by a sinusoidal oblique envelope of correlation (45° or 135°) using a blending parameter, *M*, at a frequency, $f_{m}$, 1/4 time the frequency of the carrier (Fig. 1*C*, top row; Eq. 2), as follows:

MC(x)=12(1 + M sin(2πfmx))

(2) MLE(x)=MRE(x)=12(1−M sin(2πfmx)).

The stimulus images (I_LE_ and I_RE_) presented to the two eyes were generated by blending the modulated carrier C_C_ to C_LE_ or C_RE_, respectively (Fig. 1*C*, bottom rows; Eq. 3), as follows:

$$I_{\text{LE}}=C_{C}M_{C}+C_{\text{LE}}M_{\text{LE}}$$

(3) $$I_{\text{RE}}=C_{C}M_{C}+C_{\text{RE}}M_{\text{RE}}.$$

When *M* was null, two eye images were blended with a correlated and an uncorrelated pattern equally, resulting in a partially (50%) correlated stimulus over the whole area. The correlation in the correlated stripe increased with an increasing modulation parameter and the correlation in the correlated stripe could be 100% at maximum modulation (*M* = 1), thereby making the stripes defined by the interocular correlation difference clearly visible.

### Procedure

In the binocular phase combination task, during which we measured binocular balance, participants were asked to perform an alignment (fusion) task first and then to complete a phase assessment task, during which participants’ two eyes were dichoptically presented with two horizontal gratings with opposite phase shifts (+22.5° and −22.5°). They were asked to adjust the vertical position of a 1 pixel reference line to denote where they perceived the middle of the darkest strip of the fused grating. The reported vertical location enabled us to compute the perceived phase of the binocularly perceived horizontal grating. To avoid potential positional errors, two configurations in which the phase-shift of the input was reversed were used. These two configurations were repeated eight times in each measurement session and were randomized in different trials. The perceived phase was calculated based on the 16 trials.

In the interocular correlation test, the participants’ task was to identify the orientation of the correlation envelope or the disparity corrugation (45° or 135°). In each trial, the participants were asked to press left or right keys on the keyboard to give feedback after a 1 s presentation of the stimulus. Then the qCSF method was used to obtain a full interocular correlation sensitivity function. This function can be described by the following four parameters: the peak gain, the peak frequency, the bandwidth, and the truncation. The final interocular correlation sensitivity function was picked from 100 trials with stimulus spatial frequency varying from 0.94 to 2.54 c/d. The unit employed to express spatial frequency is the number of cycles that fall within one degree of visual angle.

### Data analysis

One-way repeated-measures ANOVA was used to compare the changes of the perceived phase at different time sessions. Two-way repeated-measures ANOVA was used to show the changes of the interocular correlation sensitivity across different time sessions and to analyze the interaction relationship between the interocular correlation sensitivity and spatial frequency. We further calculated the area under the log interocular correlation sensitivity function (AULIOCSF) to investigate the magnitude of changes of the interocular correlation sensitivity over time and performed a one-way repeated-measures ANOVA for further analysis. To supplement this, we also used a Bayesian statistical approach to explore whether the nonsignificant results is a result of the true-null hypothesis (Lakens et al., 2020; Kruschke, 2021). The Bayes factor (BF_01_) is a ratio of likelihood between the null and alternative hypotheses. It can be interpreted as anecdotal at <3, as substantial at 3–10, as strong at 10–30, as very strong at 30–100, and as decisive evidence at >100 (Jeffreys, 1961) for the null hypothesis. The classical hypothesis test analysis was performed in IBM-SPSS 23.0 (IBM), and the Bayes factors were performed in JASP 0.16.1 with a default prior width (JASP team).

## Results

### The effect of 1 h alternate monocular deprivation (7 Hz) on binocular balance

**Figure 2.**
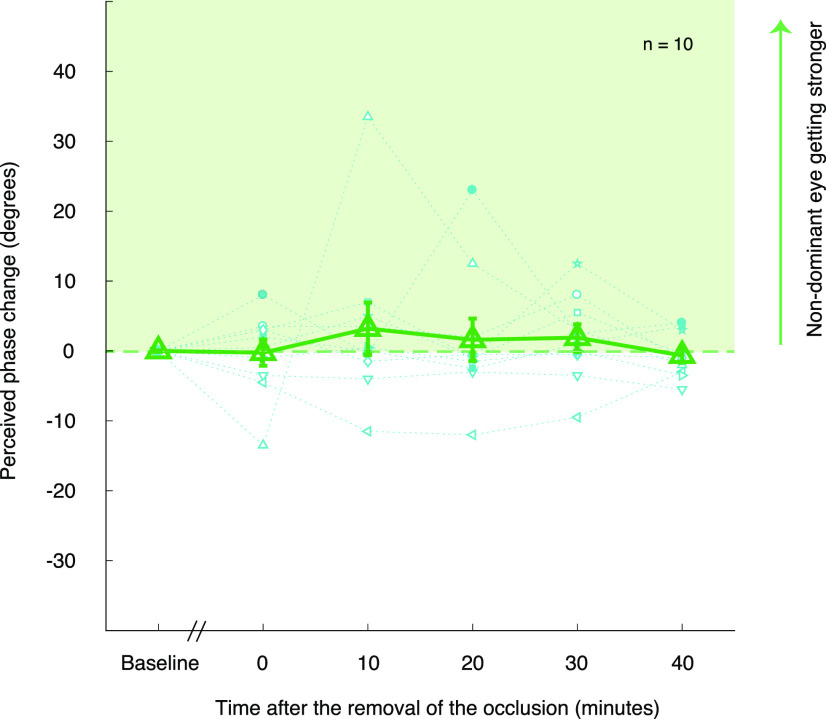
Effects of 7 Hz alternate monocular deprivation on binocular perceived phase. Results of 10 subjects’ perceived phase changes (relative to the baseline) were plotted using 10 different blue symbols, and their average perceived phase changes as a function of measurement sessions are plotted with the green triangles. Error bars represent SEs.

In this experiment, we measured the effects of short-term rapid alternate monocular deprivation on binocular balance. We used the binocular phase combination task to measure the relative contributions to binocular vision of changes in the left and right eye. The average perceived phases for 10 subjects were 0.15 ± 3.19 (before deprivation measurement; mean ± SD), −0.10 ± 6.78 (0 min after deprivation), 3.35 ± 10.54 (10 min after deprivation), 1.75 ± 7.97 (20 min after deprivation), 2.05 ± 6.25 (30 min after deprivation), and −0.50 ± 5.01 (40 min after deprivation). Regarding the data of pre-deprivation as the baseline, we found there was a little variance among four post-deprivation measure sessions. These results are displayed in Figure 2, which indicates normalized data (each postdeprivation data point subtracted by the predeprivation baseline). We conducted a one-way repeated-measures ANOVA (one within subject: time of measurements before and after deprivation sessions) to check whether the changes of the perceived phase induced by 1 h of alternate monocular deprivation was significantly different compared with the one measured predeprivation. One-way repeated-measures ANOVA showed that the change of the binocular perceived phase was not statistically significant among the time points after deprivation (*F*_(2,18)_ = 0.513, *p* = 0.611). The results of the ANOVA were complemented by a Bayesian one-way repeated-measures ANOVA. The Bayes factor was BF_01_ = 8.315, which indicates substantial evidence for the null hypothesis. In short, no significant difference in binocular balance was found between before and after deprivation.

### The effect of 1 h alternate monocular deprivation (7 Hz) on interocular correlation

**Figure 3.**
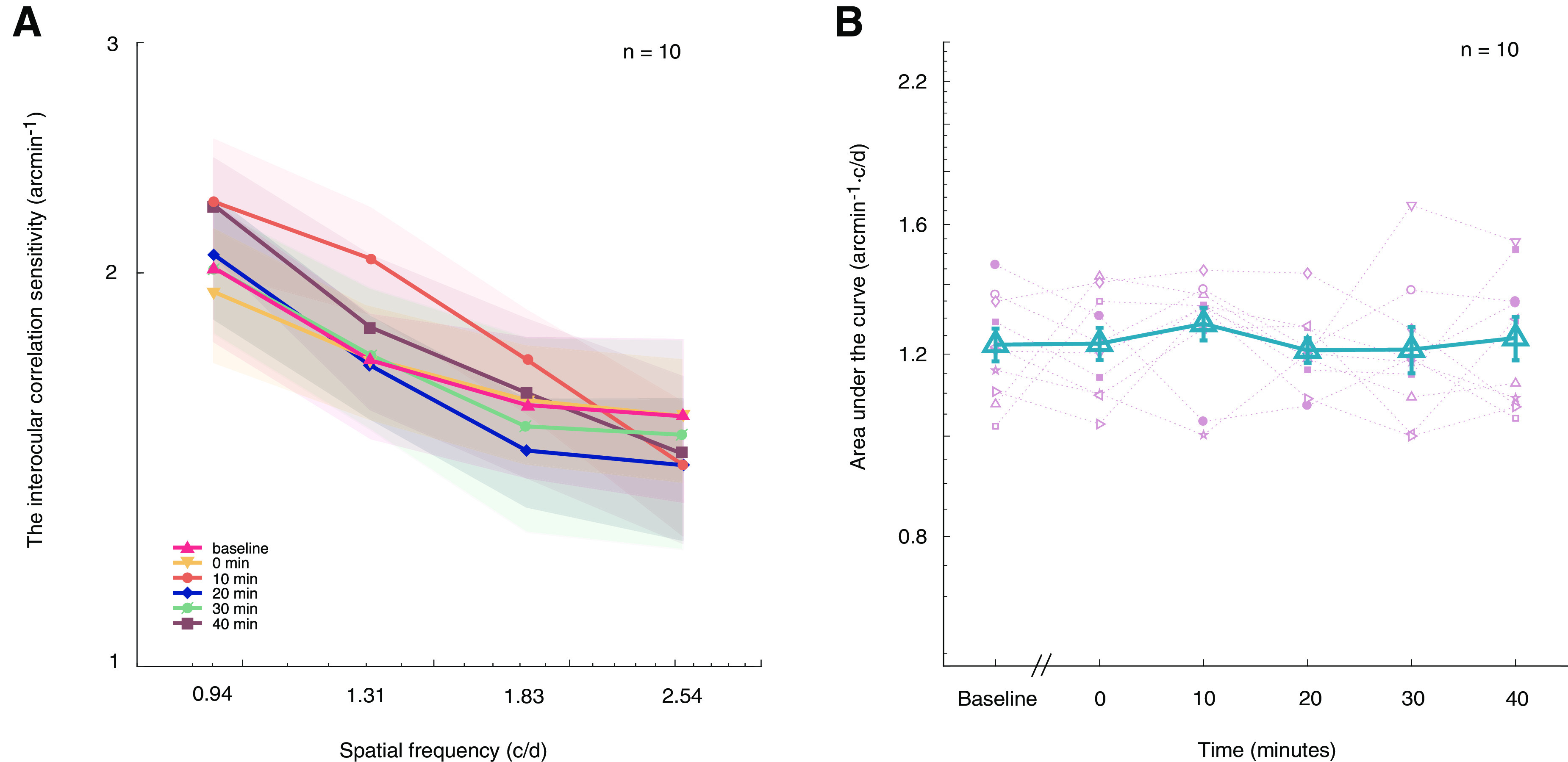
Effects of 7 Hz alternate monocular deprivation on interocular correlation. ***A***, Lines with six colors and six symbols (▵, ▿, ○, ◊, ∅, □) represent the averaged interocular correlation sensitivity as a function of spatial frequency in predeprivation and postdeprivation sessions, the shaded area indicates standard errors. ***B***, AULIOCSF in the function of predeprivation and postdeprivation sessions. Results of 10 subjects were plotted using 10 different pink symbols, and the blue triangles represent the averaged AULIOCSF across 10 subjects. Error bars denote SEs.

In the second experiment, we measured the effects of short-term rapid alternate monocular deprivation on the observer’s sensitivity to interocular correlation as a measure of low-level binocular sensitivity. In Figure 3*A*, we plotted the observers’ interocular correlation sensitivity as a function of spatial frequency at pre-deprivation (baseline) and different post-deprivation time sessions (0, 10, 20, 30, and 40 min after deprivation). The interocular correlation sensitivity functions appear to overlap before and after deprivation. Two-way repeated-measures ANOVA showed that the interaction of spatial frequency with time has no significant effect on an observer’s interocular correlation sensitivity (*F*_(3.87,34.76)_ = 0.826, *p* = 0.514), and that the change of interocular correlation was not significantly different across time (*F*_(5,45)_ = 0.361, *p* = 0.873). We further calculated the AULIOCSF and plotted the results as the function of premeasurement and postmeasurement sessions in Figure 3*B*. Indeed, there was a minimal change in the AULIOCSF for interocular correlation sensitivity. A one-way repeated-measures ANOVA showed that the effect of time on AULIOCSF was not significant (*F*_(5,45)_ = 0.361, *p* = 0.873). The Bayes factor was BF_01_ = 10.815, which is strong evidence for the null hypothesis. This finding suggests no significant effect of time on interocular correlation.

## Discussion

In this study, we investigated whether a short-term balanced disruption to binocular function could disrupt interocular balance or intrinsic binocular sensitivity (i.e., interocular correlation). Our results show that 1 h of 7 Hz flicker does not affect binocular balance and interocular correlation in normal adults.

Previous studies consistently show that depriving one eye of visual input (i.e., an unbalanced binocular disruption) shifts ocular dominance in favor of the previously deprived eye (Lunghi et al., 2011; Zhou et al., 2013b; Bai et al., 2017). A balanced disruption of the kind used here does not produce any short-term disruption to binocular function either in its binocular balance or interocular correlation sensitivity. Previous studies show that the interocular correlation sensitivity is significantly (though a little bit weakly) correlated with stereoscopic vision. These suggest that balanced deprivation using the alternate monocular deprivation might not have an obvious impact on stereoacuity. A previous study (Alanazi, 2016) has reported a decline in stereopsis after normal adults have worn the Eyetronix flicker glasses. The main difference between our study and the unpublished observations of Alanazi lies in the time points of the measurement. Specifically, our study focuses on the aftereffect of alternate deprivation while the previous study examines more on the immediate effect of the alternating stimulation. We speculated that even when stereoacuity could have been affected by the alternate deprivation with the flicker goggles, it would return to its normal state after the deprivation. On the other hand, although processing for disparity relies on interocular correlation, to the extent that if correlation sensitivity is reduced so too must be disparity sensitivity, the reverse is not necessarily true as disparity processing is a separate cortical computation.

How could we explain this lack of effect? First, it is possible that 1 h of balanced binocular disruption is not sufficiently long to produce a measureable effect. This has been extensively studied for the case where the deprivation is unbalanced through monocular deprivation. For instance, Min et al. (2018) found that 15–180 min of monocular deprivation results in substantial alterations in ocular dominance. Kim et al. (2017) also observed that monocular deprivation for 15 min could significantly increase the mean phase duration of the deprived eye. With 10 h of monocular deprivations, Ramamurthy and Blaser (2021) found that the sensory eye balance shifted away from the nondeprived eye during the first 5 h of monocular deprivation, but shifted back toward the nondeprived eye later. In our study, we applied the alternate monocular deprivation for 1 h, but it is possible that balanced deprivation requires a much longer period than its unbalanced counterpart. Second, 7 Hz alternate flicker does not abolish all binocular function; it merely reduces it (Alanazi, 2016) because of the fact that although the flicker itself is square wave and perfectly dichoptic, the visual persistence (Wilson, 1981) will afford a degree of binocular overlap. It is possible that the alternation was too rapid for any interocular suppression to occur because interocular suppression rather than simply the lack of a continuous binocular input is a more important factor. Interocular suppression in normal, and amblyopes are known to take time to develop (Huang et al., 2012; Zhou et al., 2014a, b) on the order of 150 ms, which is much longer than that produced by our rapid alternation at 7 Hz (i.e., 71 ms). Moreover, Zhou et al. (2014a, b) found that interocular suppression in amblyopic subjects and the dichoptic masking in normal subjects are because of changes in interocular contrast gain control. There is evidence suggesting that the interocular contrast gain control could be more effective with a longer time course of stimulus, which could be explained by a contrast gain control model with the contrast gain in each eye determined by the stimuli presentation duration (Ding and Sperling, 2006). We thus speculate that, during the alternate monocular deprivation, the interocular contrast gain in two eyes might be in a dynamic balance since the presentation of the stimulus in two eyes is the same. The introduction of a delay during which there is bilateral deprivation that is equivalent to the visual persistence between left and right eye stimulation might be a more exacting test of the hypothesis. Moreover, although we only recruited 10 normal adults in our study, we do not believe that this sample size was too small to show a robust result. The aim of this study was to investigate whether a short-term balanced disruption of binocular function could have the same effect as an imbalanced deprivation (e.g., monocular deprivation) because of short-term neuroplasticity. Previous studies using a binocular phase combination task similar to that used in the current study have shown that a significant change in ocular dominance could be induced after short-term monocular deprivation for a group of 6–10 normal adults (Zhou et al., 2017a, b; Min et al., 2018). Based on these studies, we also estimated the sample size via power analysis. We found that the minimum sample size of seven subjects is sufficient to achieve a power >80% based on the effects reported in the study by Min et al. (2018), and the minimum sample size of three subjects is sufficient to achieve a power >80% based on the effects reported in the studies by Zhou et al. (2017a, b). So, our decision to test 10 normal subjects is a reasonable sample size based on previous studies. Furthermore, in this study, we used the frequency of 7 Hz to measure the effect of balanced deprivation. Previous studies have shown that both short-term and long-term deprivation involving 7 Hz alternate deprivation have influences on visual function. For example, Schor and Peterson (1976) found that 7 Hz is the most effective frequency in alternation to promote visibility temporarily. Vera-Diaz et al. (2016) found that 7 Hz alternate monocular deprivation in the long term could significantly improve amblyopia reading symptoms, which also suggests that 7 Hz alternate deprivation may have had an influence on reducing suppression. However, Alanazi (Alanazi, 2016) found opposite results showing a reduction in the reading rate and stereopsis in normal adults while wearing the Eyetronix flicker glass. Though these studies have yielded varying results, they do suggest that 7 Hz is an effective frequency of alternation for inducing perceptual changes. In our study, 7 Hz alternate monocular deprivation does not have any effect on binocular balance or interocular correlation. Future studies could use slower flicker frequencies, which could be more pertinent to the phenomenon of interocular suppression.

The aim of the present research was to examine whether a balanced deprivation of binocular function would result in a significant shift in either binocular balance or intrinsic sensitivity reflecting a neuroplastic change. The results of this investigation show that 1 h alternate monocular deprivation at 7 Hz does not produce significant changes in the binocular balance or interocular correlation sensitivity of normal adults. In short, it seems that ocular dominance plasticity is unique in so far as it is driven only by an input imbalance not by balanced deprivation of binocular function at least at this alternation of deprivation.

## Synthesis

Reviewing Editor: Satu Palva, Helsingin yliopisto Decisions are customarily a result of the Reviewing Editor and the peer reviewers coming together and discussing their recommendations until a consensus is reached. When revisions are invited, a fact-based synthesis statement explaining their decision and outlining what is needed to prepare a revision will be listed below. The following reviewer(s) agreed to reveal their identity: NONE.

Monocular occlusion can cause binocular impairment (amblyopia) when administered during a critical period of development, and it is the primary method for treating amblyopia. Understanding how monocular occlusion affects binocular vision is therefore an important issue in basic and clinical vision science. Traditionally when used for treatment the better eye is occluded forcing use of the affected eye. However in recent years it has been reported that occluding the affected eye also leads to short term changes in binocularity. In this paper the authors examine whether 1 hour of rapid alternating occlusion affects binocularity. 10 normally-sighted observers wore 7Hz Eyetronix glasses for 1 hour. Their binocular vision was measured immediately before (baseline) and at 10 minute intervals after the alternating occlusion with a Binocular Balance task and an Interocular Correlation task. The results showed no significant difference between baseline and post occlusion binocular vision. The authors conclude that rapid alternation occlusion does not induce short-term binocular plasticity. The mechanisms underlying these changes are still under investigation, so the current manuscript has the potential to contribute to our understanding of the processes of short-term neuroplasticity, and also the clinical treatment of amblyopia.

The manuscript is clearly written and the experiments appear to have been conducted carefully and the data analyzed appropriately. However, the authors do not develop a theoretical argument that short-term 7Hz alternating occlusion would be expected to change binocularity in healthy subjects. Most explanations of occlusion-based plasticity are based on gain change following sustained attenuation of the input to one eye is needed. Previous researchers have demonstrated that such changes can be induced with as little as 15 minutes of occlusion. However, 7Hz alternating occlusion is equivalent to only 142 msec per eye - the authors should provide a compelling argument to justify why any binocular change would be expected under these conditions.

Major

While the Binocular Balance paradigm is well established, the Interocular Correlation sensitivity paradigm is less well understood. Moreover, interocular correlation sensitivity is only weakly correlated with stereoacuity. The authors should justify the use of this paradigm more clearly and speculate whether stereoacuity might be affected by rapid alternating occlusion. The current study investigated the possible influence of short-term balanced disruption to binocular function on interocular balance and intrinsic binocular sensitivity. The results revealed no significant differences across the key independent variables.

For the experimental design, it is crucial to ensure that the design has enough statistical/design power to tackle the target question. Thus, the study needs to prove that the sampling size is large enough, maybe via Gpower analysis, and to add a control training group in experimental design (maybe in different switch frequency? or just an unbalanced disruption) to exclude many possible alternative explanations for the current results, e.g., the history, the habituation effect.

For the statistical methods, the authors tried to use p > 0.05 to back up the null effect conclusions. Unfortunately, it has been demonstrated by a lot of statistical or psychological methods papers that this logic is very inappropriate. It is strongly suggested to use the Bayes factors and equivalence tests to inferences about null effects [Lakens et al., 2020, J Gerontol B Psychol Sci Soc Sci 75(1):45-57; John Kruschke, 2021, Nature human behavior, 1282-1291 ].

Eyetronix glasses have been used for amblyopia therapy, with mixed success, on the basis that 7Hz flicker might overcome chronic suppression of the amblyopic eye. The authors should explain how the mechanisms of suppression differ from the mechanism of gain change and how each is affected by alternating occlusion. The authors should also clarify, hat were observers performing during the 1 hour Eyetronix wearing? Was there a near vision activity, mobility activity, passive viewing or active gaming etc?

Please identify and connect the individual subject data points in the results figures so the reader can observe any consistent behavior of each subject.

For the Binocular Balance paradigm, the authors should report the matching phase as well as the phase change.

The results in figure 2 seem to show a large increase in the range of phase difference between 10-30 minutes, with a smaller range after 40 minutes.

Minor issues:

1) The monitor used in the interocular correlation measurements (ASUS PG278QR) is NOT a LED screen. Instead, it’s a traditional LCD monitor.

2) It is better to describe more in detail the information of the monitors, e.g., the displayed model of the monitor (OD, ULMB, or Gsync) as one of the previous studies has demonstrated that the temporal-spatial characters are quite different across them.
